# Dry Machining of Inconel 713LC: Surface Integrity and Force Response to Cutting Conditions

**DOI:** 10.3390/ma18173992

**Published:** 2025-08-26

**Authors:** Michal Slaný, Jan Mádl, Zdeněk Pitrmuc, Jiří Sommer, Ondřej Stránský, Libor Beránek

**Affiliations:** 1 Faculty of Mechanical Engineering, Czech Technical University in Prague, Technická 4, 160 00 Prague 6, Czech Republic; jan.madl@fs.cvut.cz (J.M.); zdenek.pitrmuc@fs.cvut.cz (Z.P.); jiri.sommer@fs.cvut.cz (J.S.); ondrej.stransky@fs.cvut.cz (O.S.); libor.beranek@fs.cvut.cz (L.B.); 2 HiLASE Centre, Institute of Physics of the Czech Academy of Sciences, 252 41 Dolní Břežany, Czech Republic

**Keywords:** nickel, Inconel, milling, dry milling, machining, surface integrity

## Abstract

While the machining of Inconel 718 has been widely studied, its cast counterpart Inconel 713LC remains underexplored, despite its relevance in high-temperature aerospace and energy components. This work presents a comprehensive investigation of dry milling behavior in Inconel 713LC, focusing on the interplay between tool wear, cutting forces, surface integrity, and chip formation across a broad range of cutting parameters. A stable process window was identified: 30–50 m/min cutting speed and 0.045–0.07 mm/tooth feed, where surface roughness remained below Ra 0.6 µm and tool life exceeded 10 min. Outside this window, rapid thermal and mechanical degradation occurred, leading to flank wear beyond the 550 µm limit and unstable chip morphology. The observed trends align with those in Inconel 718, allowing the cautious transfer of established strategies to cast alloys. By quantifying key process–performance relationships and validating predictive models for tool life and cutting forces, this study provides a foundation for optimizing the dry machining of cast superalloys. The results advance sustainable manufacturing practices by reducing reliance on cutting fluids while maintaining surface and dimensional integrity in demanding applications.

## 1. Introduction

Nickel-based superalloys are widely used in aerospace and energy applications due to their excellent mechanical and thermal properties, particularly at elevated temperatures [1,2]. Inconel 713LC, a cast Ni-Cr-Mo alloy, combines high-temperature strength with corrosion resistance, making it ideal for turbine blades and hot-section components. However, its poor machinability resulting from high strength, low thermal conductivity, and abrasive carbide phases leads to severe tool wear and elevated cutting forces [3,4]. Compared to the more widely studied Inconel 718, Inconel 713 and its variants allow for higher operating temperature, allowing for greater thermomechanical efficiencies in applications such as jet engines or energy turbines. The thermal-based benefits are exchanged for generally worse machinability. In industrial practice, the Inconel alloys are most commonly used in the as-cast condition, where critical connection features are often finished by grinding or broaching [5]. Although reliable, these processes are limited in geometric flexibility and process efficiency. Alongside the need to improve material performance in demanding applications, engineers must also find ways to lower the environmental footprint during the whole lifecycle of any part. Such considerations can include optimization of cutting conditions, cutting tool geometry or reduction, or complete elimination of cutting fluid during the machining process. Alongside environmental considerations, the machinability of Inconel 718 continues to be challenged by geometrically complex parts and variable loading conditions. The flexibility of end milling overturning makes it the preferred process in manufacturing turbine blades, thin-walled casings, and other precision components. Liao et al. [6] studied end milling of Inconel 718 and identified a stable speed window of 90–110 m/min for slot milling and 55–135 m/min for side milling. They explained this “sweet spot” by the thermal stability of γ′ precipitates, where moderate softening of the workpiece reduces forces and prolongs tool life. At higher speeds, however, excessive temperatures caused chip welding and tool softening, especially in slot milling with poor chip evacuation. They also found that feed had little influence compared to cutting speed, and that tool geometry, such as helix angle, strongly affected tool survival. Maiyar et al. [7] optimized end milling parameters for Inconel 718 to minimize surface roughness and maximize MRR using a Taguchi L9 orthogonal array combined with Grey Relational Analysis (GRA). Their results showed that the cutting speed was the most influential factor, followed by feed, while depth of cut played only a minor role. With the optimal parameters (75 m/min, 0.06 mm/tooth, 0.4 mm depth), surface roughness improved by about 10%, and the material removal rate increased by nearly 65%. This multi-response approach demonstrated the usefulness of statistical optimization tools for balancing productivity and quality in machining nickel-based superalloys.

While extensive research has been conducted on wrought superalloys such as Inconel 718 [8] or similar super alloys, studies on cast grades like Inconel 713LC remain limited, especially under dry cutting conditions. One of the rare studies was provided by Kurniawan et al. [4], who investigated the machinability of Inconel 713C and its modified compositions (T1 and T2) in turning with a WC–TiAlN coated tool. They reported that the base alloy exhibited poor machinability due to built-up edge (BUE) formation, high cutting temperatures, and catastrophic tool wear. In contrast, the modified variants showed significantly lower cutting forces, reduced tool wear, and more stable chip formation. Diffusion and abrasive wear were identified as the dominant wear mechanisms, with oxidation also contributing at higher speeds. These findings underline that even small compositional modifications can strongly affect the machinability of cast nickel superalloys.

The complex interaction between cutting conditions and material response has prompted numerous experimental and simulation-based studies aimed at improving the machinability of Inconel 718. Researchers have examined the influence of tool geometry, edge preparation, and coolant strategies on tool wear and surface integrity. For example, Outeiro et al. [9] analyzed residual stresses during dry turning, demonstrating how tool coating and cutting parameters affect stress distribution near the surface. They found that machining Inconel 718 with uncoated tools produced higher surface tensile stresses, while coated tools shifted stresses deeper below the surface, showing a strong link between tooling choice and surface integrity. Similarly, Attia et al. [10] reported that optimal parameters can simultaneously improve material removal rates and reduce cutting forces. A study on dry turning parameters on Inconel 601 was provided by Jovicic [11]. Umbrello et al. [12] investigated surface integrity in dry machining of Inconel 718, demonstrating that higher feed and cutting speed promote severe plastic deformation and up to 35% subsurface hardening. More recently, Zhou et al. [13] confirmed through hybrid modeling and experiments that chamfer geometry and feed rate strongly affect the depth of work-hardened layers, even if peak hardness remains constant [14]. The machinability of Inconel 718 thus continues to be challenged by geometrically complex parts and variable loading conditions. In the context of the surface integrity of Inconel 718, several authors highlighted the importance of optimizing machining conditions to control residual stress, surface roughness, and microhardness. Ramanujam et al. [15] used statistical models to identify dominant process parameters and employed genetic algorithms to optimize cutting speed and feed with respect to multiple performance criteria [9,16]. Thakur et al. further explored the effect of dry machining and high-speed conditions, observing that aggressive conditions often result in poor surface quality and accelerated tool wear. Studies by Venkatesan et al. [17] highlighted the benefits of coated tools in delaying failure, while Liu et al. [18] demonstrated that Ru-doped TiAlN coatings enhance hardness and stability, improving tool life by ~32%.

Cutting tool coating technology continues to evolve, with a focus on tailoring surface layers to match the thermal and mechanical demands of nickel-based alloys. TiAlN-based coatings, in particular, are recognized for their excellent oxidation resistance and high thermal stability. The incorporation of yttrium into these coatings not only enhances oxidation behavior but also refines grain structure, increasing overall toughness [18,19,20,21]. Moser et al. [22] demonstrated that such additions improve thermal stability and delay phase decomposition at elevated temperatures. Other studies have shown that dopants like molybdenum, ruthenium, and nickel contribute to enhanced coating adhesion, wear resistance, and chemical inertness during high-speed milling [18,23,24].

The sustainability of machining processes involving Inconel 718 has also garnered increasing attention in recent years. Given the high energy consumption and environmental impact of traditional cooling methods, alternative lubrication and cooling techniques such as minimum quantity lubrication (MQL), cryogenic cooling, and hybrid approaches have been extensively studied [16,25,26,27,28,29]. Shokrani et al. [16] showed that a hybrid CryoMQL technique nearly doubled tool life and reduced surface roughness by 18% compared to MQL alone, highlighting its potential for improving both efficiency and sustainability in aerospace machining.

A significant body of work has emphasized the critical role of tool coatings not only in wear mitigation but also in maintaining surface integrity and subsurface microstructure. The use of nanocomposite and multilayer coatings—including TiAlSiN, AlCrN, and TiAlMoN—has been associated with improved hardness, fracture resistance, and heat dissipation [18,19,23,24,30,31,32]. Studies by Choi et al. [33] and Aninat et al. [20] further demonstrate that doping with rare-earth elements like yttrium and tantalum enhances both oxidation resistance and chemical stability during high-speed machining.

In the context of machining performance, the surface integrity of higher-demanding Inconel 713LC after grinding has been studied by Čapek et al. [34], who reported a thin deformed layer with compressive residual stresses and fine crystallites, indicating surface hardening relevant for turbine service life.

However, challenges with machining Inconel alloys remain. Many studies to date have focused on turning rather than milling, despite the latter’s growing industrial relevance. Milling introduces interrupted cutting and higher mechanical loading, making tool failure more likely and more complex. As Alauddin et al. [35] and Sousa et al. [30] emphasized, reliable prediction of wear in these conditions requires advanced multiphysics models and coating technologies, with HiPIMS-deposited TiAlSiN showing superior tool life and oxidation resistance compared to conventional coatings. Furthermore, the synergistic effects of tool material, coating, cutting environment, and workpiece geometry are not yet fully understood, especially when extended to hybrid manufacturing or additive–subtractive combinations [36,37].

In summary, the dry milling of Inconel 713LC can be expected to involve rapid tool wear, pronounced work hardening of the subsurface, and unstable chip formation outside of limited process windows. Based on prior results in Inconel 718, stable operation is likely confined to moderate cutting speeds and feeds, while higher parameters accelerate thermal and mechanical degradation. This study therefore aims to identify those critical windows and quantify the trade-offs between tool life, surface integrity, and process efficiency in cast superalloys.

This study presents the first comprehensive analysis of the dry milling behavior of Inconel 713LC, a cast nickel-based superalloy with limited machinability data, despite its critical role in high-temperature aerospace and energy applications. Unlike prior work focused on turning or wrought grades like Inconel 718, this work systematically maps the influence of cutting parameters on tool wear, cutting forces, surface roughness, chip morphology, and subsurface hardening under dry conditions. A full-factorial design experiment is employed to derive predictive models for tool life and cutting force, supported by chip compression ratio analysis and microhardness profiling. These results directly address the lack of machining strategies for cast superalloys and enable informed process optimization for geometrically complex turbine components where grinding or broaching are insufficient.

## 2. Materials and Methods

The material used was Inconel 713LC from a single melt. The chemical composition was verified using X-ray fluorescence spectroscopy (DELTA from InnovXsystems, Woburn, MA, USA), summarized in Table 1.

**Figure 1:**
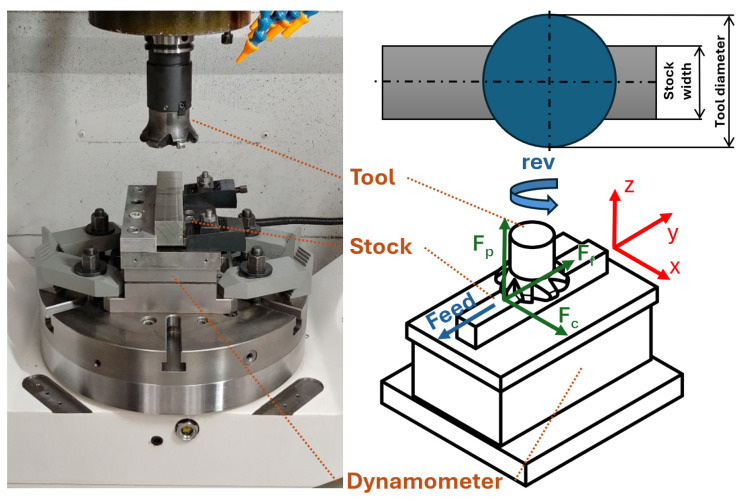
Experimental setup.

A cast and ground block with dimensions 195 × 38 × 35 mm was used for testing. Sampled surfaces of 20 mm length were milled on the block. The block was mounted on a Kistler dynamometer, aligned longitudinally with its *X*-axis and the *Y*-axis of the milling machine, as presented in Figure 1. An 80 mm diameter R200-068Q27-12M milling head from Sandvik Coromant (Sandviken, Sweden) was used with a single-coated carbide round insert RPHX 1204M4EN-M31 CTC 5240 (Ceratizit, Mamer, Luxembourg) to ensure consistent chip load. The depth of a cut was kept a constant 0.5 mm for all measurements. Dry milling was employed throughout the experiment. The main variable was cutting speed (v_c_), ranging from 11 to 100 m/min. Feed per tooth (f_z_) ranged from 0.02 to 0.12 mm/tooth. For the force measurement, the full-factorial experiment has been chosen. The depth of cut was the same for all tests, set at 0.5 mm.

A full-factorial design experiment was employed in order to capture both main effects and parameter interactions across the full range of cutting speeds and feeds. This approach was chosen over Taguchi or RSM methods, as the goal of this study was comprehensive mapping of machinability rather than parameter optimization.

For force analysis, milling experiments were performed on a vertical machining center Okuma MU-400 (Ōguchi, Japan), and the cutting forces were recorded using a Kistler (Winterthur, Switzerland) dynamometer system:

- Dynamometer: Kistler 9265B
- Amplifier: Kistler 5019
- A/D Converter: Kistler 5697
- Sampling rate: 5000 Hz
- Force limits: F_x_, F_y_ = 2000 N, F_z_ = 2500 N

Flank wear (VB) was measured using an optical microscope VHX-6000 from Keyence (Osaka, Japan). Tool life was defined according to ISO 3685:1993 [38], using a flank wear criterion of VB = 0.55 mm, which in our experiments marked the onset of rapid wear progression and loss of machining stability. For each cutting condition, up to three trials were performed.

Chip samples were collected manually, and their maximum thickness h_c_ was measured using a micrometer to calculate the chip compression ratio (CCR) as h_c_/h_0_, where h_0_ is the undeformed chip thickness computed from feed, engagement, and tool geometry.

Surface roughness was measured using a handheld contact roughness tester, Mahr Marsurf PS10 (Göttingen, Germany), with three scans per surface. The roughness was evaluated using Ra and Rz parameters, which are standard in machining studies. While Ra represents the mean surface profile and is commonly used for general quality assessment, Rz captures the maximum peak-to-valley height and is therefore more sensitive to local irregularities such as built-up edge or rapid tool wear effects that may not be evident in Ra.

Hardness was measured via the microhardness measuring device FM-100, from Future-Tech (Kawasaki, Japan) utilizing the standard procedure defined by ISO 6507-1:2023 [39] HV0.3 with 300 g of force load. The machined surface was slant cut under a 5-degree inclination to allow measuring microhardness at the distance 0.077 mm from the top surface and then with an increment of 0.095 mm. Each depth layer was measured at 8 different places and averaged.

Metallographic etching was used for optical microstructure analysis with the optical microscope Keyence VHX-6000, with objective VH-ZST.

For detailed surface and subsurface morphology evaluation, selected cross-sections were further analyzed using a scanning electron microscope, JEOL 7600F (Tokyo, Japan). SEM was employed to detect fine features such as deformation bands, grain boundary distortion, microcracks, and carbide morphology near the machined surface. Observations focused on areas within 0.1 mm of the surface to evaluate the depth and nature of machining-induced deformation under varying cutting conditions.

## 3. Results and Discussion

### 3.1. Tool Wear

**Figure 2:**
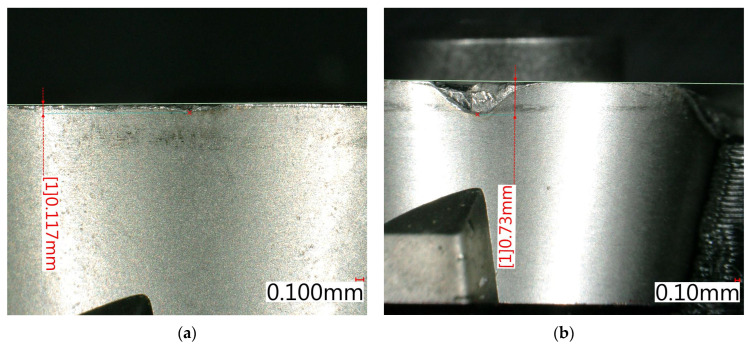
Examples of wear. (**a**) v_c_ = 50 m/min, f_z_ = 0.02; (**b**) v_c_ = 70 m/min, f_z_ = 0.12.

Tool wear evaluation was focused on identifying critical cutting conditions leading to excessive flank wear or catastrophic tool failure. The limit for acceptable wear was set to VB = 0.55 mm, based on measured values. After this threshold, wear continued in a very rapid manner. Representative examples of measured flank wear are presented in Figure 2.

As shown in Table 2, no significant tool wear was observed at cutting speeds up to 50 m/min across all tested feed rates. However, at cutting speeds of 70 m/min and above, combined with feed rates ≥ 0.045 mm per tooth, rapid wear progression resulted in exceeding the wear limit and complete tool edge failure during machining. This trend highlights the sensitivity of Inconel 713LC milling to thermal and mechanical loads under dry conditions, emphasizing the need to limit cutting speeds in combination with higher feeds to avoid premature tool failure. The predominant wear mechanisms included abrasive wear combined with plastic deformation caused by heat and edge overload.

Those findings confirm similar patterns reported in milling of Inconel 718 [40]. Higher feed rates accelerated wear progression due to increased chip thickness, although the specific cutting resistance slightly decreased.

To better capture the influence of feed on tool durability, tool life was estimated using a wear-based model where VB measurements were divided by the time per cut and scaled to a failure threshold. The results are listed in Table 3.

For instance, at v_c_ = 70 m/min, f_z_ = 0.095 mm/tooth, tool wear progressed to ~420 µm in only 0.59 min of cutting, corresponding to an estimated tool life of ~1 min. This sharp contrast with the ~35 min observed at v_c_ = 11 m/min under the same feed demonstrates the exponential impact of thermal loading on carbide tool life.

### 3.2. Estimated Taylor Model of Tool Life

The Taylor relation was chosen for tool life estimation because of its simplicity, robustness, and widespread industrial use. It captures the effect of cutting speed (and here also feed) with minimal data requirements, making it suitable for initial predictive modeling. Its main limitation is that it does not explicitly include other variables or complex wear mechanisms, and extrapolation beyond the tested range is not recommended. More advanced models may improve accuracy, but at the cost of greater data requirements and complexity.

(1) $$T=108.65\cdot v_{c}^{-1.70}\cdot f_{z}^{-1.25},$$

A Taylor-type model was fitted to the estimated tool life data using nonlinear regression. The resulting relationship showed excellent agreement with an R^2^ of 0.9979. The Breusch–Pagan test (*p* = 0.68) indicated homoscedastic residuals with constant variance, while the Shapiro–Wilk test (*p* = 0.0012) revealed slight deviations from normality. These deviations occurred mainly at extreme combinations of low feed and low speed, where experimental scatter is typically highest. Despite this, the model provides robust predictive capability within the tested domain and confirms the strong dependence of tool life on both cutting speed and feed.

The results revealed that although high feed values increased the mechanical load, the shorter engagement time per surface partially compensated for this effect. These findings are consistent with the literature, where higher feed rates increase cutting temperature and stress, yet tool life is affected by the interaction between thermal and mechanical loads, as shown in Inconel 718 and AISI 304 machining [40,41].

Based on the data, the recommended process window for tool life can be determined for T > 10 min: v_c_ = 30–50 m/min, f_z_ ≤ 0.07 mm/tooth.

### 3.3. Forces

**Figure 3:**
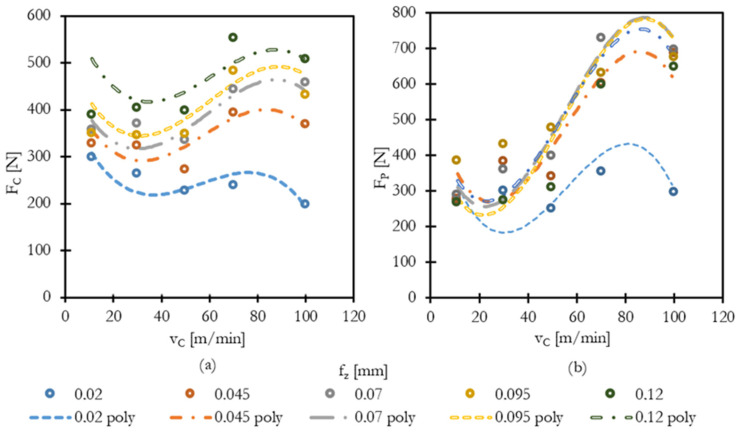
(**a**) Cutting force as a function of cutting speed and (**b**) passive force as a function of feed per tooth.

The measured forces during cutting were evaluated in terms of the cutting force (F_C_) and passive force (F_P_). The influence of cutting speed (v_c_) and feed per tooth (f_z_) on force development was analyzed. The numerical results of the measurement are presented in Table 4 (Cutting Forces) and Table 5 (Passive Forces). The data is visually presented in Figure 3. As expected for nickel-based superalloys, both force components exhibited sensitivity to changes in cutting conditions.

Selected force measurements are summarized in the following tables, with graphs provided to better illustrate the observed trends.

At low cutting speeds (v_c_ = 11–30 m/min), forces remained relatively stable, with moderate increases linked to higher feeds. However, at cutting speeds above 50 m/min, a nonlinear escalation of forces was observed, particularly for the passive force (F_P_), indicating increased tool wear and possible instability due to thermal effects and edge degradation.

A polynomial regression model was developed to describe the relationship between cutting parameters and the cutting force. The best fit with acceptable model complexity was achieved using a third-degree (cubic) polynomial, which captures linear, quadratic, and interaction effects while avoiding excessive overfitting:

(2) $$\begin{array}{cc} F_{C}=342-14.2 & \cdot \;v_{c}+5450\cdot f_{z}+0.26\cdot v_{c}^{2}+64.5\cdot v_{c}\cdot f_{z}-83000\cdot f_{z}^{2}\\ & -0.00156\cdot v_{c}^{3}+0.219\cdot v_{c}^{2}\cdot f_{z}-545\cdot v_{c}\cdot f_{z}^{2}+482000\cdot f_{z}^{3} \end{array}$$

The developed polynomial regression model for F_c_ yielded an R^2^ of 0.906, supporting its predictive capability within the tested parameter range. Residual analysis confirmed no systematic bias, and a Shapiro–Wilk test (*p* = 0.77) indicated that the residuals follow an approximately normal distribution. The Breusch–Pagan test (*p* = 0.14) did not indicate significant heteroskedasticity, suggesting constant variance across the tested domain.

(3) $$\begin{array}{cc} F_{P}=469.083\;+ & 6759\cdot f_{z}-31.53\cdot v_{c}-148100\cdot f_{z}^{2}+209\cdot f_{z}v_{c}+0.6336\\ & \cdot v_{c}^{2}+786100\cdot f_{z}^{3}-1108\cdot f_{z}^{2}v_{c}-11.99\cdot f_{z}v_{c}^{2}-0.003780\\ & \cdot v_{c}^{3} \end{array}$$

Similarly, this polynomial regression model for F_p_ yielded an R^2^ of 0.825, supporting its predictive capability within the tested parameter range. Residual analysis of the polynomial model confirmed no systematic bias, and a Shapiro–Wilk test (*p* = 0.74) indicated that the residuals follow an approximately normal distribution. The Breusch–Pagan test (*p* = 0.09) was slightly below the conventional 0.1 threshold, suggesting a possible trend toward heteroskedasticity, although not statistically strong. Overall, the third-order model can be considered adequate within the tested domain. Nevertheless, testing a fourth-order polynomial improved statistical indicators markedly (R^2^ = 0.984, adj. R^2^ = 0.961) and resolved the heteroskedasticity issue, while maintaining normally distributed residuals. This suggests that higher-order polynomials or alternative regression forms may better capture the observed nonlinearities, but at the cost of increased model complexity and reduced robustness outside the tested parameter window.

The models fitted to the force data exhibit a local maximum, which is likely an artifact of the polynomial fitting rather than a true mechanical transition. Due to the chosen model form, the regression models are valid only within the tested parameter window, and extrapolation outside this range is not recommended. Care should be taken in interpreting such mathematical features.

Figure 3a shows the development of the cutting force F_c_ with increasing cutting speed for various feed rates combined with the polynomial regression model. A moderate increase in F_c_ is visible with higher feeds, while cutting speed has a less pronounced effect up to 50 m/min. Beyond this threshold, a noticeable rise occurs, particularly at maximum feed values.

Figure 2b highlights the passive force F_p_, where a sharp increase is evident at higher cutting speeds, especially for f_z_ ≥ 0.07 mm/tooth. This suggests that the possible impact of combined rapid tool wear and complicated chip formation contributes significantly to force escalation.

The cutting force Fc ranged from 197 N (v_c_ = 100 m/min, f_z_ = 0.02 mm/tooth) up to 552 N (v_c_ = 100 m/min, f_z_ = 0.12 mm/tooth). The passive force F_p_ increased even more steeply, from 273 N to 857 N across the same range, indicating a rising tool load and deformation resistance with increasing feed and cutting speed.

Forces of cutting analysis revealed that both the cutting and the passive force increased with feed and cutting speed. However, force growth was more sensitive to feed per tooth than to cutting speed and had a stronger and more linear influence. This is consistent with the fact that feed directly affects chip thickness and the material removal rate.

The strongest influence was observed with feed per tooth: increasing f_z_ from 0.02 to 0.12 mm/tooth at v_c_ = 50 m/min led to a nonlinear rise in F_c_, possibly due to significant edge wear. This highlights the mechanical load impact of chip volume per tooth. High F_p_ values suggest increased friction, rubbing, and potential instability at elevated speeds and feeds.

The decline in the forces of cut at higher cutting speeds (the upper range of tested conditions) can be explained by two main factors.

First, at low feed rates, the reduction in force is likely attributed to thermal softening. A small feed produces a thin chip with a high chip compression ratio, which concentrates mechanical work into a smaller deformation volume. This leads to more heat being dissipated in a localized region of the chip, softening the material ahead of the tool and thus lowering resistance to cutting. Second, at higher feed rates, the situation becomes more complex. Although increasing feed generates more heat—potentially enhancing thermal softening—the combined effect of elevated temperature and mechanical load leads to accelerated tool wear. Edge wear effectively reduces the real depth of cut and the load-bearing area of the tool, while simultaneously altering the tool geometry. This change increases friction and the tendency for force escalation, despite thermal effects.

These trends confirm the behavior typical for machining nickel-based superalloys under dry conditions, where excessive cutting speeds lead to instability and higher mechanical load on the tool.

It should be noted, however, that the decrease–increase–decrease trend in the regression curve (Figure 3) is partly a mathematical artifact of polynomial fitting. Polynomial models of second and higher order can introduce local extrema that are not directly present in the raw experimental data. The overall trends (thermal softening at low feeds, force escalation due to tool wear at higher feeds) are physically valid, but the apparent secondary decrease should not be over-interpreted as a distinct mechanical phenomenon.

The polynomial regression models confirm that force behavior is nonlinear but predictable. Therefore, these models can be a useful tool for force estimation, machine load planning, and integration into simulation tools. Cutting force data provides a basis for future tool wear and energy consumption modeling. It can be used for predicting deflection in the machine–tool–workpiece system to maximize the accuracy of production by selecting proper tools and toolholders and designing sturdy workholding. Findings support a recommended process window of v_c_ = 30–50 m/min, f_z_ = 0.045–0.07 mm/tooth for the stability and tool life for the tested tool and tool insert.

The rise in both cutting and passive forces at higher feeds is directly linked to increased mechanical load and flank wear (see Section 3.1), which in turn shortens tool life, as captured by the Taylor model (Section 3.2).

### 3.4. Surface Roughness

The influence of cutting speed and feed per tooth on surface roughness parameters Ra (arithmetically mean roughness) and Rz (maximum height of profile) was evaluated under dry milling conditions.

**Figure 4:**
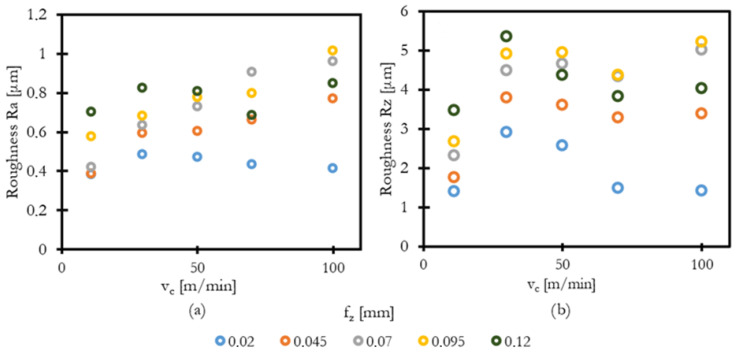
Surface roughness: (**a**) Ra (arithmetic mean roughness) and (**b**) Rz (maximum height of profile).

Figure 4a,b illustrate the influence of cutting speed and feed per tooth on surface roughness parameters Ra and Rz during milling of Inconel 713LC. Both parameters of roughness present a nonlinear but structured trend. This behavior cannot be described as a simple function of feed and tool shape but is more complex and is at least v_c_ dependent.

The lowest Ra values were observed at the lowest cutting speed 11 m/min. The Rz values show a general trend across all feeds, where the lowest cutting speed produced the lowest roughness proportional to feed. Higher speed produces a steep rise in Rz roughness between 30 and 50 m/min, presumably caused by an unstable build-up edge randomly creating raised sharp bumps on an otherwise smooth surface. For higher speeds, Rz drops for most feeds, because the tool is leaving the zone of BUE formation. The high speed and high feed combinations are generally worse, caused by rapid tool wear, and therefore dependent on irregularities of the worn-out edge. The difference between Ra and Rz parameters shows that the machined surface is mostly smooth, but it is stained by irregular peak spots.

For the best surface finish, low cutting speed can be recommended. Generally, avoiding the area of unstable BUE formation makes sense for avoiding random surface irregularities. High cutting speed cannot be recommended because rapid tool wear changes the tools’ edge.

### 3.5. Chip Formation

In nickel-based superalloys, chip formation is typically characterized by segmented or saw-tooth chips, resulting from localized shear deformation and thermal softening in the primary shear zone. This phenomenon, known as adiabatic shear banding [41,42,43], is influenced by cutting conditions and material properties. The chip compression ratio (CCR), defined as the ratio of deformed chip thickness to undeformed chip thickness, serves as an indicator of plastic deformation during machining.

Chip thickness measurements were conducted using a micrometer at the thickest point of each chip’s cross-section. For each set of cutting parameters, eight individual measurements were taken to account for variability in chip formation. The numerical results are presented in Table 6.

**Figure 5:**
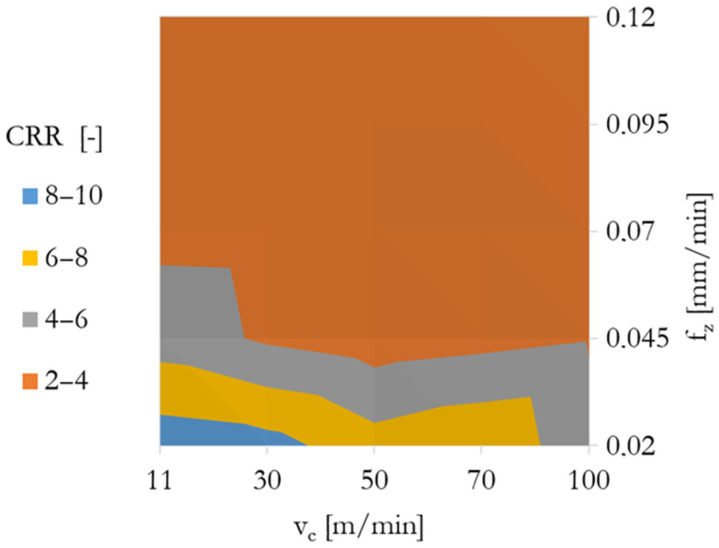
Chip compression ratio graph as a function of cutting speed v_c_ and feed per tooth f_z_.

The measured chip compression ratios (CCRs) ranged from over 9 at low feeds and speeds (f_z_ = 0.02 mm/tooth, v_c_ = 11 m/min) down to values near 2 at the highest cutting regimes. This indicates a significant reduction in plastic strain with an increasing material removal rate. High CCR at low v_c_/f_z_ indicates severe plastic deformation and inefficient chip separation, what could possibly lead to high surface hardening. The values are visually presented in Figure 5.

High CCR values reflect intense shear deformation and limited chip separation, correlating with higher measured surface hardness in the subsurface layer. In contrast, lower CCR at high feeds and speeds suggests more efficient chip formation, though it also correlates with higher tool wear and thermal load. These findings are in agreement with typical CCR trends for nickel-based superalloys under dry machining conditions [44].

The CCR range confirms that at low cutting conditions, plastic deformation is high and chip removal inefficient, which coincides with increased subsurface work hardening. On the other hand, lower CCR values at a higher feed and speed indicate improved chip separation, albeit with greater tool wear.

The correlation between CCR and measured flank wear supports the hypothesis that excessive plastic strain in the chip zone directly contributes to increased mechanical and thermal stress on the tool edge.

**Figure 6:**
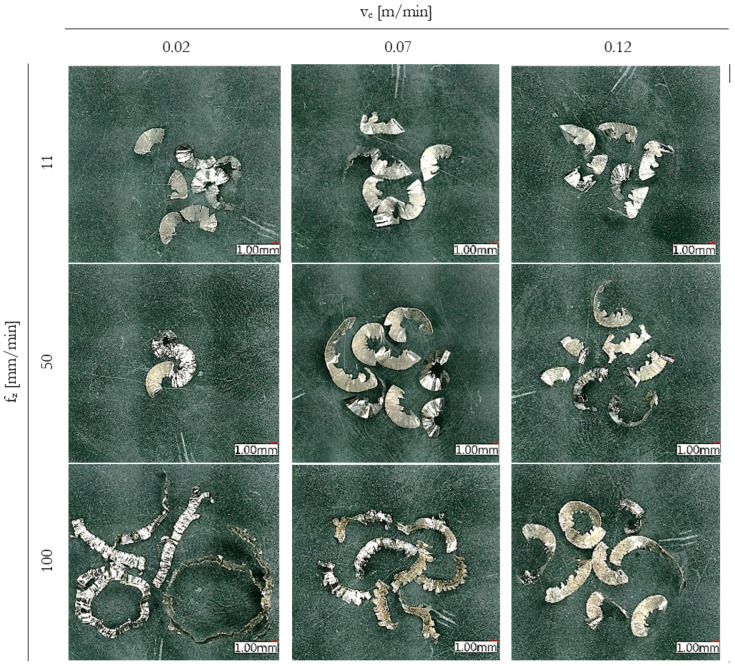
Chip shape comparison with increasing cutting speed v_c_ and feed per tooth f_z_.

The observed chip morphology transitioned from short, compact segments at low cutting speeds to longer, curled or strip-like chips at higher speeds, especially at v_c_ = 100 m/min, as presented in Figure 6. At low feeds, a thin chip appeared to remain on the tool edge, potentially remaining to follow up cut and fusing with a chip from another layer. Those chips are therefore longer than they should be.

At the lowest feed and cutting speed (f_z_ = 0.02 mm/tooth, v_c_ = 11 m/min), chips were extremely compact, with an average thickness exceeding 0.07 mm, which is more than 3.5× the undeformed chip thickness. These chips had a flattened, blocky appearance and were frequently broken into short segments, consistent with intense plastic deformation and poor chip evacuation.

In contrast, at higher cutting conditions (f_z_ = 0.12 mm/tooth, v_c_ = 100 m/min), chips were thinner relative to the undeformed thickness (CCR ≈ 2.1) and exhibited more regular, curled shapes. This indicates a transition toward thermally assisted shear and more effective chip formation, despite the increased thermal load observed in tool wear data.

With increasing feed per tooth, a more pronounced jagged profile developed along the inner side of the chip, indicating intensified shear instability and higher chip thickness. These features align with typical chip segmentation patterns reported in Inconel 718 milling [6,26] suggesting the applicability of the Inconel 718 machining experience towards Inconel 713LC.

### 3.6. Surface Hardening

Forces induced by machining of the previous layer cause plastic deformation in the newly generated surface, which results in surface hardening. This machining-induced work-hardening phenomenon has been widely reported for nickel-based superalloys, both on machined surfaces [12,13,14] and in chips [44]. Various materials react differently, depending on their microstructure and strain-hardening capacity.

Those effects were tested on both examined nickel alloys. Examined were just single cutting parameters for a_p_ and f_z_, the same as they were in the previous tests and dry surrounding conditions.

**Figure 7:**
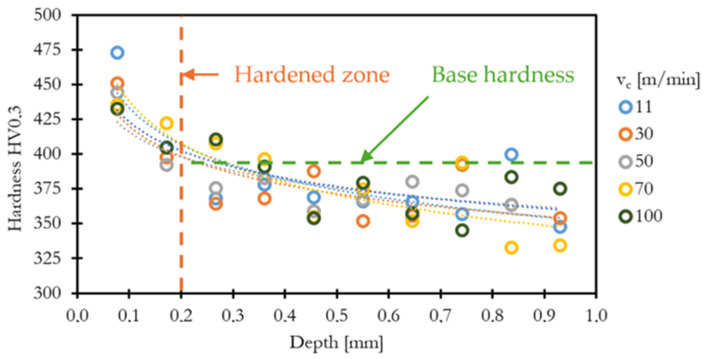
Hardness changes in a surface layer.

The microhardness measurements presented in Figure 7 indicate a significant influence of cutting speed on the surface integrity of machined Inconel 713LC. At a cutting speed of 11 m/min, the surface hardness reached approximately 473 HV0.3, suggesting substantial work hardening due to severe plastic deformation, corelating with chip formation and CCR findings.

The hardened layer extends up to 0.2 mm beneath the surface, gradually decreasing to the base material hardness (~350 HV). At higher cutting speeds, such as 100 m/min, the surface hardness decreased to around 433 HV0.3, likely due to thermal softening effects. Compared to Inconel 718, the increase in surface hardness of Inconel 713LC was less pronounced, with differences below 100 HV, whereas machining of Inconel 718 often results in work hardening exceeding 100 HV above the base material [12,13,14]. However, the depth of the hardened layer was comparable, remaining clearly detectable up to 0.1–0.2 mm beneath the machined surface.

Microstructural observations were conducted using optical microscopy on metallographically prepared cross-sections of the machined samples.

**Figure 8:**
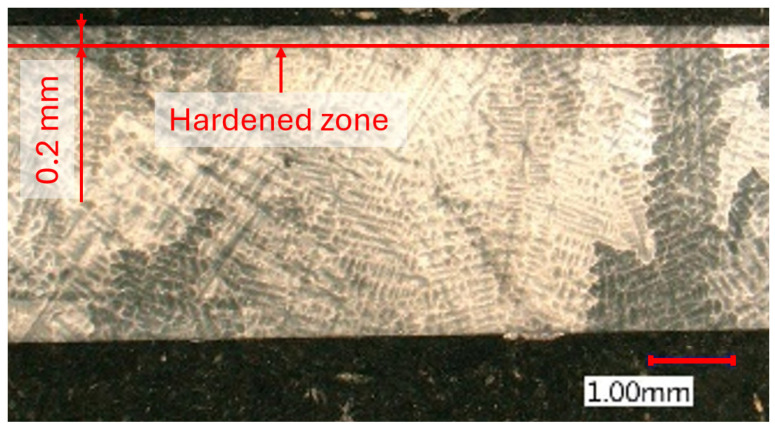
Microstructure of as-cast workpiece.

The etched microstructure of Inconel 713LC presented in Figure 8 revealed a typical dendritic morphology characteristic of cast nickel-based superalloys. No signs of recrystallization or phase transformation were observed in the bulk structure, indicating that dry milling did not alter the overall metallurgical stability of the material.

**Figure 9:**
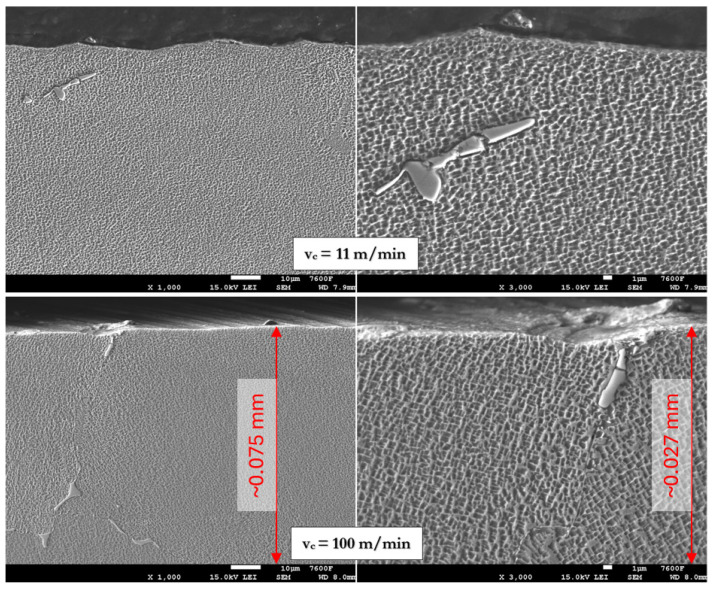
SEM surface layer cross-section using 1000× magnification (**left**) and 3000× magnification (**right**) for the lowest cutting speed (**top row**) and highest cutting speed (**bottom row**).

In Figure 9, the as-cast microstructure of Inconel 713LC can be seen, showing a γ/γ′ matrix typical of nickel-based superalloys. The γ phase forms the continuous matrix, while the γ′ precipitates appear as bright cuboidal particles (visible as holes in this type of etching). Their heterogeneous distribution reflects solidification in the as-cast state.

MC-type carbides can be seen segregated in the intracrystalline regions, consistent with the as-cast condition. Their morphology and distribution remain unchanged after machining, confirming that dry milling did not alter the carbide population or overall microstructural stability of Inconel 713LC.

A close-up of the surface layer did not show any eminent in microstructure morphology, as apparent from Figure 9. Highly deformed matrix should show temperature-related changes. None of those changes were clearly visible originating from the machined surface. Those deformed areas are present in the material, but they are probably the result of the previous casting process. The position and shape of the carbide inclusions also remained stable, without signs of shattering or smearing through the surface layer. These observations indicate that surface hardening originates mainly from strain-induced plastic deformation, while the overall microstructural stability suggests that dry milling preserves the metallurgical integrity of Inconel 713LC, even under elevated energy input.

The observed force trends are closely linked to tool wear progression, tool life, and chip compression behavior. At low-to-moderate cutting speeds (30–50 m/min), forces remain relatively stable due to limited flank wear, shown in the longer tool life estimated by the Taylor relation. In this range, high CCR values are obtained at very low feeds (f_z_ = 0.02 mm/tooth), reflecting severe plastic deformation and unstable chip separation, which contributes to subsurface hardening and higher surface roughness. With increasing feed and speed, flank wear accelerates and CCR decreases, indicating more efficient chip formation. This transition temporarily suppresses unstable BUE, improving the surface finish and reducing Rz. However, once wear becomes the dominant factor, forces escalate, and the surface roughness deteriorates again. Ultimately, the combined influence of CCR, cutting forces, wear mechanisms, and tool life defines the process window: beyond v_c_ ≥ 70 m/min and f_z_ ≥ 0.095 mm/tooth, rapid tool failure occurs, reducing tool life way below 1 min. These relationships confirm that plastic deformation in the chip zone, force evolution, tool degradation, and surface integrity are interdependent and must be considered together when optimizing cutting conditions.

## 4. Conclusions

This study demonstrates that dry milling of Inconel 713LC is not only technically feasible but also a promising step toward more sustainable and environmentally responsible machining of high-performance superalloys. By eliminating the need for cutting fluids, dry milling addresses growing ecological and occupational concerns—such as coolant disposal, contamination, and health risks—while simplifying process logistics in the aerospace and energy sectors. The results show that high surface quality and reasonable tool life can be achieved, but only within a narrow and well-controlled process window. Beyond this range, excessive thermal and mechanical loading leads to rapid tool degradation and poor surface integrity.

The observed cutting behavior of Inconel 713LC shows clear parallels with Inconel 718, reinforcing that insights from the more extensively studied alloy can be transferred with caution. Chip formation, wear progression, and surface hardening trends align well, with CCR highlighting the link between plastic deformation and tool wear. At low feeds, high CCR indicated severe strain and subsurface hardening, while at higher feeds and speeds, CCR decreased, reflecting more efficient chip separation but at the cost of accelerated wear. Increased cutting speed also suppressed unstable BUE and temporarily improved the surface finish, though this effect diminished as tool wear became dominant.

The originality of this study lies in providing the first systematic evaluation of dry milling of Inconel 713LC, integrating tool wear, cutting force modeling, surface integrity, and chip formation into a comprehensive process map. These contributions expand the limited machinability data for cast superalloys and support both industrial practice and future academic research.

Key Findings

- Tool wear accelerates at high speeds (v_c_ ≥ 70 m/min) and feed rates (f_z_ ≥ 0.12 mm/tooth), often leading to catastrophic flank failure; lower parameters greatly extend tool life.
- Cutting forces increase significantly with feed rate; cutting speed has a nonlinear effect, remaining stable up to ~50 m/min, but increasing sharply above 70 m/min, particularly in the passive force component.
- Surface roughness (Ra ≈ 0.4–0.6 µm) deteriorates at 30–50 m/min due to unstable chip formation and likely BUE effects.
- Chip Compression Ratio (CCR) decreases from >9 to ~2 as speed increases, strongly correlating with surface hardening and tool wear rates.
- Surface microhardness is highest at low cutting speeds, indicating significant plastic deformation and work hardening, in line with CCR observations.
- Empirical models (Taylor tool life and polynomial force models) fit well (R^2^ ≈ 0.8–0.9), offering predictive insight for process optimization within the tested parameters.

Recommendations

- For best results, maintain cutting speeds between 30 and 50 m/min and feed per tooth between 0.045 and 0.07 mm to ensure balanced performance.
- Avoid f_z_ > 0.095 mm/tooth under dry conditions, unless the productivity demand justifies increased tool consumption.

Limitations

- Tests were conducted exclusively under dry conditions; results may differ with MQL or flood cooling.
- Only uncoated tools were tested; alternative tool materials or coatings may exhibit different wear and thermal behaviors.
- In situ thermal monitoring was not included, limiting direct insight into heat-related degradation mechanisms.
- Machining lengths were limited (~20 mm), so long-term wear progression and surface evolution remain to be explored.
- Residual stress and fatigue performance were not evaluated, though observed surface hardening implies potential mechanical implications.
- The regression models are valid only within the tested range; extrapolation beyond it may introduce artifacts and requires further validation.

## Figures and Tables

**Table 1:** Chemical composition of Inconel 713LC (XRF analysis).

Element	Ni	Al	Ti	Cr	Zr	Fe	Nb	Mo
wt. %	73.87 ± 0.22	5.65 ± 0.22	0.75 ± 0.04	12.63 ± 0.08	0.091 ± 0.004	0.18 ± 0.01	2.16 ± 0.02	4.29 ± 0.02

**Table 2:** Tool flank wear.

Flank Wear [mm]		Cutting Speed v_c_ [m/min]				
		11	30	50	70	100
**Feed per tooth f_z_ [mm]**	0.02	0.04	0.08	0.12	0.19	0.46
	0.045	0.05	0.07	0.14	0.22	0.59
	0.07	0.05	0.10	0.16	0.38	0.65
	0.095	0.06	0.11	0.18	0.42	0.83
	0.12	0.08	0.17	0.36	0.73	1.56

**Table 3:** Tool life estimation.

Tool Life Estimation [min]		Cutting Speed v_c_ [m/min]				
		11	30	50	70	100
**Feed per tooth f_z_ [mm]**	0.02	247.3	45.3	18.1	8.2	2.4
	0.045	87.9	23.0	6.9	3.1	0.8
	0.07	56.5	10.4	3.9	1.2	0.5
	0.095	34.7	6.9	2.5	0.8	0.3
	0.12	20.6	3.6	1.0	0.4	0.1

**Table 4:** Cutting force results (F_C_ in N).

F_c_ [N]		Cutting Speed v_c_ [m/min]				
		11	30	50	70	100
**Feed per tooth f_z_ [mm/tooth]**	0.02	298	262	227	237	197
	0.045	328	322	272	393	368
	0.07	357	370	335	443	458
	0.095	430	345	348	483	430
	0.12	520	403	397	552	507

**Table 5:** Passive force results (F_P_ in N).

F_p_ [N]		Cutting Speed v_c_ [m/min]				
		11	30	50	70	100
**Feed per tooth f_z_ [mm/tooth]**	0.02	273	300	250	353	298
	0.045	277	382	340	600	687
	0.07	288	360	338	778	695
	0.095	303	327	337	755	675
	0.12	347	273	308	857	648

**Table 6:** Average chip thickness and chip compression ratio.

v_c_ [m/min]		11		30		50		70		100	
f_z_ [mm]	h_0_ [mm]	h_c_ [mm]	CCR [-]	h_c_ [mm]	CCR [-]	h_c_ [mm]	CCR [-]	h_c_ [mm]	CCR [-]	h_c_ [mm]	CCR [-]
0.02	0.008	0.073	9.2	0.070	8.7	0.055	6.8	0.062	7.8	0.036	4.5
0.045	0.018	0.092	5.1	0.067	3.7	0.053	2.9	0.061	3.4	0.070	3.9
0.07	0.028	0.098	3.5	0.096	3.4	0.088	3.1	0.089	3.2	0.093	3.3
0.095	0.037	0.127	3.4	0.124	3.3	0.124	3.3	0.132	3.6	0.107	2.9
0.12	0.047	0.130	2.8	0.141	3.0	0.141	3.0	0.148	3.1	0.101	2.1

## Data Availability

The original contributions presented in this study are included in the article. Further inquiries can be directed to the corresponding author.

## Abbreviations

The following abbreviations are used in this manuscript:

MQL	Minimum quantity lubrication
CCR	Chip compression ratio
BUE	Built-up edge
